# Estimating suicide occurrence statistics using Google Trends

**DOI:** 10.1140/epjds/s13688-016-0094-0

**Published:** 2016-11-08

**Authors:** Ladislav Kristoufek, Helen Susannah Moat, Tobias Preis

**Affiliations:** 1 Data Science Lab, Behavioural Science, Warwick Business School, University of Warwick, Coventry, CV4 7AL UK; 2 Institute of Economic Studies, Faculty of Social Sciences, Charles University, Opletalova 26, Prague, 110 00 Czech Republic

**Keywords:** nowcasting, search data, Google Trends, official statistics

## Abstract

Data on the number of people who have committed suicide tends to be reported with a substantial time lag of around two years. We examine whether online activity measured by *Google* searches can help us improve estimates of the number of suicide occurrences in England before official figures are released. Specifically, we analyse how data on the number of *Google* searches for the terms ‘depression’ and ‘suicide’ relate to the number of suicides between 2004 and 2013. We find that estimates drawing on *Google* data are significantly better than estimates using previous suicide data alone. We show that a greater number of searches for the term ‘depression’ is related to fewer suicides, whereas a greater number of searches for the term ‘suicide’ is related to more suicides. Data on suicide related search behaviour can be used to improve current estimates of the number of suicide occurrences.

**Electronic Supplementary Material:**

The online version of this article (doi:10.1140/epjds/s13688-016-0094-0) contains supplementary material.

## Introduction

The identification of causes of suicide attempts and suicide occurrences is a topic which has attracted the interest of a number of scientists in psychology and psychiatry [1–9] as well as in other social sciences such as demography, sociology and economics [10–16]. One of the challenges of analysing and modelling suicides from a macroscopic perspective is a long time lag in their reporting in official statistics. Identifying additional sources and data which would help estimate the number of suicide occurences before official data are available is thus of high importance and interest. In recent years, studies of the online activity of Internet users have proven fruitful in various fields ranging from medicine [17, 18], ecology [19, 20] and epidemiology [21–25] to linguistics [26], politics [27], sociology [28] and economics, finance and behavioural science [29–49]. For example, previous studies have provided evidence that online data may help us reduce delay and cost in measuring human behaviour [22, 40, 42, 43, 47], allow us to measure aspects of society and our environment that were previously difficult to measure [34, 41, 44, 45], and in some cases, even predict future actions [30, 35, 38, 39, 48, 49].

Here, we investigate whether data on searches relating to depression and suicide can help us address the problem of delayed data on suicides, and generate estimates of the number of suicide occurrences before official figures are released. A number of previous studies have attempted to investigate whether online search data might provide an avenue for creating quicker estimates of the number of suicide occurrences [50–55]. However, these analyses were subject to a number of important restrictions. For example, McCarthy [50] examined the possible link between suicide occurrences and online activity in the USA. A strong negative correlation of -0.9 was reported between the yearly number of suicide occurrences and the yearly search activity for the term ‘suicide’. This finding was, however, based on a very limited data sample only (specifically, annual data between 2004 and 2007). Page *et al.* [51] studied monthly online search activity of suicide-related search terms in Australia between 2004 and 2011. They found no evidence for a significant link to suicide rates. However, their analysis was very restricted due to the availability of suicide data in Australia. Page *et al.* therefore limited themselves to analysing seasonal patterns in search activity and its relationship to changes in unemployment, which is frequently reported to be connected to suicides rates. No connection to suicide rates or suicide statistics was thus examined. Sueki [52] analysed a monthly suicide time series for Japan between 2004 and 2009 by calculating cross-correlation coefficients. Using the terms ‘suicide’, ‘depression’ and ‘suicide method’ translated into Japanese, Sueki found that increasing numbers of suicide occurrences coincide with increased online search activity for the ‘depression’ term only. At the same time, increasing search activity for the ‘depression’ term also appeared to be linked to a decrease in the actual suicide rates three months both earlier and later. The author thus suggests that the Internet could help prevent suicides by providing meaningful information to individuals who are depressed. The relevance of the results is, however, again weakened by a limited dataset (a monthly time series from 2004 to 2009). Yang *et al.* [53] investigated monthly suicide time series for Taipei in Taiwan, covering the time period from 2004 to 2009. The authors analysed 37 suicide-related search terms and reported that searches for a number of terms could be connected to the number of suicide occurrences for specific age groups, as well as specific types of suicide. However, we note that the authors did not control for possible non-stationarity of either suicide or online search data. Hagihara *et al.* [54] studied suicide rates in Japan between 2004 and 2010 for individuals with an age between 20 and 40. Utilizing the Box-Jenkins transfer function, the authors found several positive links between online search activity and suicidal behaviour. However, considering the number of observations (77), the number of analysed terms (20), the number of lags included in the transfer functions (12) and seasonal adjustments, it is difficult to exclude the possibility that the low number of statistically significant connections at specific lags may result from statistical error. In addition, Gun III and Lester [55] carried out a cross-sectional correlation analysis of state-level data from the USA in 2009. A positive correlation was found for all three search terms which they use - ‘commit suicide’, ‘how to suicide’ and ‘suicide prevention’. However, in this final study, the authors restrict themselves to a cross-sectional analysis and do not investigate the possibility of using search data to improve estimates across time.

Even though generalisations are difficult to make based on the reviewed studies, due to difficulties with data access and the potential methodological limitations described above, the search terms ‘suicide’ and ‘depression’ seem to be leading candidates for a model of suicidal behaviour which incorporates online search data. We therefore make use of these terms in our analysis. At the same time, we avoid the methodological pitfalls identified in the previous studies. Specifically, we study monthly time series of suicide occurrences in England between 2004 and 2013, which provides enough data for reliable estimation and statistical analysis. Further, we control for specific dynamic properties of the suicide and search query data - seasonality, non-stationarity and possible lagged dependence. The dataset analysed here also makes it possible to investigate the potential for using online searches to estimate suicide incidence numbers in practice, before the official data arrives. We refer to this as a ‘nowcasting’ analysis, in which we are ‘predicting the present’ [40].

## Methods

### Data

We study monthly suicide occurrence statistics in England between 2004 and 2013 provided by Office for National Statistics (ONS, www.ons.gov.uk).^1^ These data are made available with a pronounced lag of approximately 24 months. Suicide numbers are given for both males and females and different age brackets. Due to the coarseness of the data, we conduct our analysis on the overall occurrences, but do not investigate differences between gender and age groups.

Previous studies have suggested that searches for the terms ‘suicide’ and ‘depression’ may relate to real world suicide rates. We obtain data on the number of *Google* searches made for these terms from the website *Google Trends* (trends.google.com). Data are retrieved from *Google* at monthly granularity and relate to searches made in England only. The number of queries for a given term is rescaled to a value between 0 and 100. This holds for all search data retrieved from *Google Trends*, potentially weakening the value of *Google* data in modelling, as the actual number of searches is not provided. However, compared to other alternatives such as *Twitter* or *Wikipedia* data, *Google* search data provide much longer time series with easy geographical localisation. Both these characteristics are crucial for our analysis.

### Models

As a benchmark model for suicide occurrences, we use a simple autoregressive model with seasonal dummy variables

1 $$ \mathit{SUI}_{t}=\alpha_{0}+ \alpha_{1}\mathit{SUI}_{t-24}+\sum _{m=1}^{11}\mu _{m}M_{m,t}+ \varepsilon_{t}, $$

where $\mathit{SUI}_{t}$ represents the number of suicide occurrences in month $t=25,\ldots,T$. We use a lag of 24 months to account for the fact that suicide data is released with two years delay. Variables $M_{m,t}$ are dummy variables equal to one if the observation at time *t* is the specific month *m*, and zero otherwise.

A competing model utilizing *Google* search queries is specified as follows

2 $$\begin{aligned} \mathit{SUI}_{t} =&\beta_{0}+ \beta_{1}\mathit{SUI}_{t-24}+\sum_{j=0}^{q=12}{ \delta_{j}\mathit{DEPRESSION}_{t-j}} \\ &{}+\sum_{j=0}^{q=12}{\zeta_{j} \mathit{SUICIDE}_{t-j}}+\sum_{m=1}^{11} \mu _{m}M_{m}+\nu_{t} \end{aligned}$$

for $t=25,\ldots,T$ and a lag order *q* is set equal to 12 months. This allows us to control for annual seasonalities, and also enables us to investigate the relationship between *Google* search volume and the number of suicides at a range of different monthly lags. $\mathit {DEPRESSION}_{t}$ and $\mathit{SUICIDE}_{t}$ are monthly *Google* queries for the respective terms.

Multicollinearity issues and a high number of regressors might make this estimation procedure unstable. A higher number of variables increases the variance of the estimators so that the results are less reliable. To address this problem, we use the Almon distributed lag model [56] which reduces the number of estimated parameters. The model is based on a flexible approximation of a dynamic relationship between dependent and independent variables using the polynomial lag structure. Setting the number of lags according to Eq. (2) equal to $q=12$ and choosing a quadratic polynomial^2^ ($p=2$) as an approximation of possible dynamic relationship between the number of suicides and related *Google* search queries, we can rewrite Eq. (2) as

3 $$\begin{aligned} \mathit{SUI}_{t} =&\eta_{0}+ \eta_{1}\mathit{SUI}_{t-24}+\sum_{j=0}^{q} \Biggl(\mathit{DEPRESSION}_{t-j}\sum_{w=0}^{p=2}{ \iota_{w}j^{w}} \Biggr) \\ &{}+\sum_{j=0}^{q} \Biggl( \mathit{SUICIDE}_{t-j}\sum_{w=0}^{p=2}{ \kappa _{w}j^{w}} \Biggr)+\sum_{m=1}^{11} \mu_{m}M_{m}+u_{t}. \end{aligned}$$

This reduces the number of estimated parameters from $2*(q+1)+13$, i.e. 39 in our case, in Eq. (2) to $2*(p+1)+13$, i.e. 19 in our specific case, in Eq. (3). Note that 13 out of these account for an intercept, lagged suicide occurrences, and seasonal dummy variables. We obtain estimates of the original model in Eq. (2) via a transformation of the estimates from Eq. (3) as

4 $$ \begin{aligned} &\hat{\delta}_{j}=\sum_{w=0}^{2}{ \hat{\iota}_{w}j^{w}}, \\ &\hat{\zeta}_{j}=\sum_{w=0}^{2}{ \hat{\kappa}_{w}j^{w}}. \end{aligned} $$

This specification is robust to multicollinearity between dependent variables. By introducing a dependency structure into the setting, it allows for further interpretation of the relationship between the examined variables.

### Model testing and performance

We apply a standard set of tests during the estimating procedure. First, we test whether the model would benefit from adding polynomial (usually squared and cubic) transformations of the dependent variables, using the Ramsey’s RESET test [57]. If we reject the null hypothesis of the test, the model should be re-specified with further variables. Second, we run tests to ensure that the variance of the error terms is not unevenly distributed, or heteroskedastic, as this makes statistical tests less efficient. We use the ARCH effect test [58] to test for heteroskedasticity. To deal with static heteroskedasticity, we employ heteroskedasticity and autocorrelation consistent standard errors [59]. Third, to seek further evidence that the model is well specified, we test for normality of residuals using the Jarque-Bera test [60]. This test is less essential as rejecting normality of residuals usually does not have any serious consequences for the estimated model. However, not rejecting normality is usually taken as a sign of a very well specified and functional model. Fourth, we investigate whether the parameters of our model change across time using the CUSUM test [61]. If the null hypothesis is not rejected, the estimated model is considered stable in time. We test for significance of separate regressors using a *t*-test, and joint significance using an *F*-test. In both cases, to avoid problems which could be caused by autocorrelation and heteroskedasticity, we use robust standard errors.

The quality of the estimated models is inspected using the coefficient of determination $R^{2}$ and the adjusted coefficient of determination $\bar{R}^{2}$, which controls for a number of independent variables. To give a further metric of the quality of the estimations made by the model, we also compare mean absolute percentage error (MAPE) for competing models. A higher MAPE indicates that a model is making lower quality estimates. MAPE is defined as

5 $$ \mathit{MAPE}=\frac{100}{N}\sum_{t\in\mathbb{T}}{\biggl\vert \frac{\mathit {SUI}_{t}-\widehat{\mathit{SUI}}_{t}}{\mathit{SUI}_{t}}\biggr\vert }, $$

where $\widehat{\mathit{SUI}}_{t}$ is the fitted value of suicide occurrences, $\mathbb{T}$ is an interval over which the model is estimated and treated, and *N* is a number of observations in $\mathbb{T}$.

### Nowcasting performance

The relationship we are investigating here is of most interest due to potential practical exploitation, where *Google* search data could be used to estimate the number of suicide occurrences in the past month, before the official counts arrive. Such estimates are often referred to as ‘nowcasts’ [40], as the goal is not to forecast future values of a time series, but to estimate the value of the time series for the current period, drawing on past values of the time series and other relevant indicators. Estimates of these kinds are often constructed using standard forecasting methods.

We note that while finding a model that can describe the time series well is of value, good explanatory power does not necessarily imply that the model can be used to make estimates in practice. This is particularly true for models of non-stationary and seasonal time series, which can deliver very good fits but only poor forecasting performance. For this reason, we carry out a separate analysis to determine the nowcasting performance that can be achieved by including *Google* search data.

## Results

### Basic analysis

**Figure 1:**
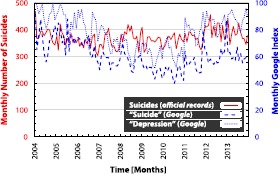
**Official data on suicide occurrences and** ***Google*** **searches for ‘depression’ and ‘suicide’.** We analyse monthly data from official records of suicide occurrences in England (left *y*-axis). We investigate whether monthly data on *Google* searches for ‘depression’ and ‘suicide’ in England (right *y*-axis) can help estimate counts of suicide occurrences before the official data are available. Note that search data retrieved from *Google* are normalised to create an index which takes integer values between 0 and 100. Higher values indicate that a higher proportion of the total searches in England in a given month were for the term of interest.

We study how the number of suicide occurrences in England changes over time, and how these changes may be reflected in the online activity of individuals. Due to data availability, we restrict our analysis to the years 2004-2013. We illustrate the monthly suicide statistics in Figure 1. Full descriptive statistics are provided in Table 1. The number of suicide occurrences remains stable in time, with a mean of 370 per month. We find no evidence of non-normal distribution of the data (Table 1) but relatively strong serial correlation structure (Table 2). To test for stationarity and the presence of unit roots, we use the Augmented Dickey-Fuller (ADF) test [62] and the KPSS test [63] with a maximum lag of three months. Finding evidence of unit roots would make cointegration or transformation of our data necessary [64]. We find evidence of no unit roots for the suicides data, although the KPSS test leads us to reject the null hypothesis of stationarity for this time series (Table 2). Given the conflicting results of these tests, we proceed to study the suicide time series in its original form, but perform additional out-of-sample testing later on to help verify that any non-stationarity has not led to misleading results.

**Table 1:** **Descriptive statistics of data on suicide occurrences**

	**Mean**	**SD**	**Min**	**Max**	**Skewness**	**Ex. kurtosis**	**Jarque-Bera**	***p*** **-value**
Suicides	370.20	20.65	302	468	0.3797	0.3328	3.4377	>0.1

**Table 2:** **Autocorrelation and unit-root tests**

	***Q*** **(12)**	***p*** **-value**	**ADF**	***p*** **-value**	**KPSS**	***p*** **-value**
Suicides	41.9279	<0.01	−5.4869	<0.01	0.4956	0.0451
Google						
-*Depression*	496.0180	<0.01	−2.3241	>0.1	0.7141	0.0131
-*Suicide*	410.2039	<0.01	−1.2876	>0.1	1.2190	<0.01

The *Q*-test checks for autocorrelation in the first 12 lags of the series. We find evidence of autocorrelation for the *Google* data as well as for the suicide series. The ADF test has a null hypothesis of the presence of unit roots, which is not rejected for the two *Google* time series but is rejected for the suicide time series. The KPSS test has the null hypothesis of stationarity, which is rejected for both *Google* time series and for the suicides time series.

To investigate whether data from *Google* can help us to estimate the number of suicide occurrences in England before official figures are released, we follow the findings of the previous studies and analyse data on *Google* searches for terms ‘depression’ and ‘suicide’. Figure 1 depicts the search query time series. We find that both follow a very similar pattern in time (with a Pearson’s correlation of 0.6580, $p < 0.01$). Both series are strongly autocorrelated (*Q*-test: see Table 2), and are identified as non-stationary and unit root processes (KPSS and ADF tests: see Table 2). From a methodological point of view, the presence of unit roots does not rule out a standard regression procedure, as long as both explanatory variables - in our case the *Google* searches - are unit root processes, which holds in our case [65].

Several studies have argued that *Google* data on searches for the terms we use, ‘depression’ and ‘suicide’, are correlated with data on suicide occurrences. Table 3 reports the estimated correlation coefficients between suicide occurrences and *Google* searches for these *Google* terms. In addition to the correlation previously noted between the *Google* searches, we find evidence of only a weak correlation between searches for ‘depression’ and suicide occurrences, and no correlation between searches for ‘suicide’ and the suicide data. This suggests two things. First, the information content of data on searches for the terms ‘depression’ and ‘suicide’ might be very similar. Second, there is either little value in the online data for estimating suicide rates, or the simple correlation analysis is insufficient. We therefore construct a model which goes beyond this simple correlation framework.

**Table 3:** **Coefficients for correlations between data on** ***Google*** **searches for ‘depression’ and ‘suicide’ and official data on suicide occurrences**

	**Depression (Google)**	***p*** **-value**	**Suicide (Google)**	***p*** **-value**
Suicides	0.2124	0.0198	0.1626	0.0760
Depression (Google)			0.6580	<0.01

We find that the correlation between occurrences of suicides and *Google* searches for ‘depression’ is statistically significant but low. There is no evidence of a correlation between occurrences of suicide and *Google* searches for ‘suicide’. *Google* searches for the two terms are strongly correlated.

### Models

As a base model, we create a model which controls for reported seasonal patterns in suicide occurrences and takes into account the most recent suicide statistics at our disposal. We approximate the two year lag in the release of suicide statistics for England by assuming that at each point in time, the most recent data we have is for 24 months ago. Specifically, we use a simple autoregressive model with the seasonal dummy variables specified in Eq. (1). As we are working with data at monthly frequency, monthly seasonal dummies are utilised. For the autoregressive term, we use a time lag of 24 months, to reflect the delay in data release. The ‘*Google* model’ controls for the same factors as the base model but also incorporates data on *Google* searches for the terms ‘depression’ and ‘suicide’ (Eq. (2)). Data on both terms are included at various lags, from 0 to 12 months, to account for both instantaneous as well as lagged effects. This allows us to investigate whether data on *Google* searches at different lags may help us estimate suicide rates. Such a detailed analysis has not been performed for the suicide data in the literature yet.

Table 4 summarises the important statistics of the estimated models. We observe that the base model performs reasonably well, with an $R^{2}$ of 0.23. This means that a simple seasonal model can explain 23% of the total variation in suicide occurrences. We also report the results for a model with *Google* searches only, which is referred to as the ‘control model’. The control model outperforms the base model, with an $R^{2}$ of 0.28. However, the improvement is limited.

**Table 4:** **Model quality**

	$\boldsymbol{R^{2}}$	$\boldsymbol {\bar{R}^{2}}$	**MAPE**	**RESET**	***p*** **-value**
Base model	0.2263	0.1144	5.6401	0.1712	>0.1
Control model	0.2810	0.2326	5.8724	0.4308	>0.1
*Google* model	0.4620	0.3362	4.9390	0.2473	>0.1
*Google vs Base*	+0.2357	+0.2218	−0.7011	-	-

Analyses of $R^{2}$, $\bar{R}^{2}$, and MAPE statistics all provide evidence that the base model is enhanced when data on *Google* searches for the terms ‘depression’ and ‘suicide’ are added. The RESET test results suggest that we do not need to add polynomial transformations of the dependent variables.

In contrast, the complete *Google* model (Eq. (3)), where data on online searches enrich the base model, provides a more notable improvement, leading to an $R^{2}$ of 0.46. This provides initial evidence that data on searches for these terms may help us estimate suicide rates before official data are released. Model improvement is demonstrated not only by an increase in $R^{2}$ (0.46 compared to 0.23) but also by increases in adjusted $R^{2}$ ($\bar{R}^{2}$) which accounts for the number of independent variables in the regression (0.34 compared to 0.11). Furthermore, the mean absolute percentage error (MAPE) of the model decreases from 5.64% to 4.94%.

All models pass the standard testing procedures, which are reported in Table 5 and described in more detail in the Methods section. In addition, Table 6 provides *F*-statistics and demonstrates that the data on *Google* searches add statistically significant explanatory power to the model. As a complementary analysis, we also perform a Likelihood Ratio (LR) test to compare the performance of the base model and the *Google* model including data on *Google* searches for both the terms ‘depression’ and ‘suicide’. Again, we find that the *Google* model provides a better fit to the data ($\chi^{2}=34.88$, $df=6$, $p<0.01$).

**Table 5:** **Additional tests**

	**J-B test**	***p*** **-value**	**ARCH effect**	***p*** **-value**	**CUSUM**	***p*** **-value**
Base model	1.7621	>0.1	17.6826	>0.1	4.1902	<0.01
Control model	0.9725	>0.1	11.3692	>0.1	0.7985	>0.1
*Google* model	0.2470	>0.1	9.9840	>0.1	0.2472	>0.1

The Jarque-Bera test checks normality of residuals, the ARCH effect test controls for conditional temporal heteroskedasticity in residuals and the CUSUM tests stability of the model across time. We find no evidence that the residuals are not normal, and no evidence of heteroskedasticity in the residuals. For the control model and the *Google* model, we find no evidence that the parameters change across time, although this does not hold for the base model.

**Table 6:** **Model improvement through inclusion of** ***Google*** **data**

**Depression**	***p*** **-value**	**Suicide**	***p*** **-value**	**Joint**	***p*** **-value**
4.7620	<0.01	7.9329	<0.01	5.6225	<0.01

An *F*-test provides further evidence that data on searches for the terms ‘depression’ and ‘suicide’ help explain a significant proportion of variance in the suicide data.

**Figure 2:**
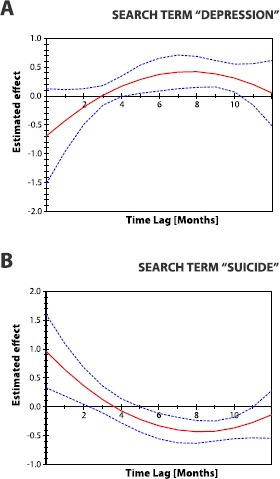
**Estimating official suicide counts using** ***Google*** **search data.** We investigate the relationship between the volume of searches for **(A)** ‘depression’ and **(B)** ‘suicide’ and the number of suicide occurrences, with a lag between the search data and the suicide data of up to 12 months (*x*-axis). Solid red lines represent the estimated effect and the dashed blue lines illustrate the 90% confidence intervals of these effects. At a lag of 0 months, we find that a higher number of searches for the term ‘depression’ corresponds to a lower number of suicides. However, this effect is not statistically significant. For lags of 5 to 10 months, a greater number of searches for the term ‘depression’ corresponds to a greater number of suicides. Conversely, at a lag of 0 months, we find that a greater number of searches for the term ‘suicide’ corresponds to a higher number of suicides. The effect vanishes after approximately 2 months. For lags of 6 to 11 months, a greater number of searches for the term ‘suicide’ corresponds to a lower number of suicides. However, it should be noted that the changes between positive and negative effects for both search terms may be due to the polynomial shape induced by the Almon model specification.

These analyses therefore provide evidence that data from *Google* can help us estimate the number of suicide occurrences in England before official figures are released. Figure 2 illustrates the interactions between the *Google* search data and data on suicide occurrences implied by the Almon model (Eq. (3)). We find that the relationship between the suicide data and search data for ‘depression’ is negative at lag zero and that it weakens with additional time lags, getting close to zero after approximately three months (Figure 2A). However, this pattern is not statistically significant. For lags of 5 to 10 months, a greater number of searches for the term ‘depression’ corresponds to a greater number of suicides. Conversely, we find that the relationship between the suicide occurrences and the ‘suicide’ search term is positive at the zero lag and the effect vanishes after approximately 2 months. For lags of 6 to 11 months, a greater number of searches for the term ‘suicide’ corresponds to a lower number of suicides. (Figure 2B). We note that the changes between positive and negative effects for both search terms may be due to the shape imposed by the Almon model specification. Note that qualitatively similar results hold even if data on the two search terms are included in the model separately, which implies that their conflicting behaviour is not caused by multicollinearity. Together, these results provide further evidence that monitoring of the number of suicide occurrences could potentially be improved using data on dynamics of the online searches, given that official suicide data for England are available only with a lag of two years.

**Figure 3:**
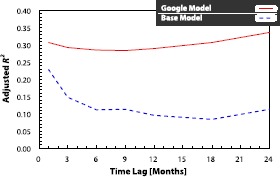
**Effect of different delays in data availability on model quality.** As the delay in availability of data on suicide occurrences varies for the public and policymakers, we perform the analysis for a range of possible delays. Specifically, we re-estimate the *Google* model in Eq. (3) using lags of 1, 3, 6, 9, 12, and 18 months, in addition to the original reporting delay of 24 months (*x*-axis). This covers delays in data availability for public (24 months delay), policymakers (around 6 months delay) and hypothetical models (a delay below 6 months). The adjusted $R^{2}$ values for the base model (dashed blue line) and the *Google* model (solid red line) with corresponding delays are shown on the *y*-axis. We observe that the results do not vary greatly when the lag in data availability is modified. The only mild deviation is observed for hypothetical delays of one or three months, where the base model improves, but in these cases too, the *Google* model still performs better than the base model. This provides further evidence of the potential value of online data in estimating suicide occurrence statistics.

As the delay varies for the public and for policymakers, we perform the analysis for various delays.^3^ Specifically, we re-estimate the *Google* model in Eq. (3) using lags of 1, 3, 6, 9, 12, and 18 months in addition to the original reporting delay of 24 months. This covers the data availability for public (24 months delay), policymakers (around 6 months delay) and hypothetical models (a delay below 6 months). The adjusted coefficients of determination $\bar{R}^{2}$ for the base and the *Google* models with corresponding delays are summarised in Figure 3. We observe that the results are very stable, and the *Google* model provides a clear improvement for all delays, including hypothetical delay lengths below 6 months. This provides further evidence of the value of the online search data in estimating suicide statistics for the most recent month.

### Nowcasting analysis

Our analysis is limited by the number of data points which overlap between the official records of the number of suicide occurrences and search data from *Google*. Data on suicides are available only at monthly granularity, with the most recent records stemming from 2013, whereas online search data are available from 2004 only. As a result, our analysis is limited to ten years of monthly data points, or 120 data points. Up to this point, the results we have reported are all drawn from ‘in-sample’ analyses, where models are fitted to the full data set. However, the question remains as to whether a relationship between online data and official statistics on suicides could be used in practice to estimate the number of suicide occurrences in the past month, before the official data are released with several months delay.

To investigate this, we perform a small nowcasting study using the available data, which as a by-product helps verify that our ‘in-sample’ results are not due to overfitting and non-stationarity of the *Google* data. In Table 7, we present statistics on the ‘out-of-sample’ performance of the *Google* model compared to the base model. Both models are fitted using data gathered between 2004 and 2011, and performance is tested for years 2012 and 2013. We find that use of the *Google* search data does indeed lead to lower errors in estimates, as evaluated using three different error metrics.

**Table 7:** **Nowcasting performance**

	**Base model**	***Google*** **model**
Mean absolute error	29.559	15.059
Root mean squared error	41.564	34.59
Mean absolute percentage error	7.728	7.125

The *Google* model leads to lower errors than the base model, as evaluated by three different error metrics.

## Discussion

Counts of the number of suicide occurrences in England are released with a delay of two years. Here, we investigate whether estimates of the number of suicide occurrences can be generated using data from *Google* searches. We find that using *Google* data, estimates of the number of suicides between 2004 and 2013 can be improved in comparison to estimates from previous suicide data alone.

Our findings are in line with the hypothesis that data on *Google* searches for ‘depression’ and ‘suicides’ may help improve estimates of the number of suicide occurrences in England before official figures are released. The results we report highlight the potential value of online communication data for creating new proxy measures of psychiatric illness across large populations.

## Electronic Supplementary Material

Below is the link to the electronic supplementary material.

**Dataset.** The dataset is provided in a comma separated value file (csv).

## Footnotes

1 Monthly suicide occurrences are available at https://www.ons.gov.uk/peoplepopulationandcommunity/birthsdeathsandmarriages/deaths/adhocs/005582numberofsuicidesbymonthofoccurrenceregionsofenglandandwales1981to2013. Occurrence counts are provided for regions of England, which we sum to get statistics for all of England. The complete dataset is attached as Additional file 1 - Dataset.

2 In our analysis, we have evaluated polynomials up to $p=4$. Selection of $p=2$ provides the most stable results.

3 Interested readers are referred to the Statistical Bulletins of ONS at http://www.ons.gov.uk for comparison of the reported delay and the actual data availability.
